# A four-methylated mRNA signature-based risk score system predicts survival in patients with hepatocellular carcinoma

**DOI:** 10.18632/aging.101738

**Published:** 2019-01-10

**Authors:** Yu Wang, Zhiping Ruan, Sizhe Yu, Tao Tian, Xuan Liang, Li Jing, Wenyuan Li, Xiao Wang, LCL Xiang, F.X. Claret, Kejun Nan, Hui Guo

**Affiliations:** 1 Department of Medical Oncology, The First Affiliated Hospital of Xi'an Jiaotong University, Xi’an, Shaanxi, PR China; 2 Department of Systems Biology, The University of Texas MD Anderson Cancer Center, Houston, TX 77030, USA

**Keywords:** score system, nomogram, prognosis, methylation, hepatocellular carcinoma

## Abstract

Evidence suggests that altered DNA methylation plays a causative role in the pathogenesis of various cancers, including hepatocellular carcinoma (HCC). Thus, methylated differently expressed genes (MDEGs) could potentially serve as biomarkers and therapeutic targets in HCC. In the present study, screening four genomics profiling datasets (GSE62232, GSE84402, GSE73003 and GSE57956) enabled us to identify a total of 148 MDEGs. A signature was then established based on the top four MDEGs (BRCA1, CAD, CDC20 and RBM8A). Taking clinical variables into consideration, we constructed a risk score system consisting of the four-MDEG signature and the patients’ clinical features, which was predictive of prognosis in HCC. The prognostic value of the HCC risk score system was confirmed using TCGA HCC samples. The scores were then used to construct a nomogram, performance of which was evaluated using Harrel’s concordance index (C-index) and a calibration curve. The signature-based nomogram for prediction of overall survival in HCC patients exhibited good performance and was superior to traditional staging systems (C-index: 0.676 vs 0.629, P< 0.05). We have thus established a novel risk score system that is predictive of prognosis and is a potentially useful guide for personalized treatment of HCC patients.

## Introduction

Hepatocellular carcinoma (HCC) is the fifth most common cancer in China, where it is estimated to have killed 140 million people [1]. The leading cause of HCC is chronic infection with a hepatitis virus, alcohol abuse, exposure aflatoxin, tobacco smoking and diabetes [2]. Although there are a large number of studies examining HCC formation and progression, the precise mechanism underlying its pathogenesis remains unclear [3]. Moreover, the rate of early diagnosis of HCC is low; most patients are diagnosed with advanced disease. TNM stage at diagnosis is still regarded as the best predictor of survival [4]. However, because HCC is a highly heterogeneous malignancy, the prognoses of patients with the same stage disease may differ due to inherent clinical and molecular diversities [5]. Therefore, new valid and reliable prognostic and predictive biomarkers are needed to improve risk prediction and offer better information for guiding personalized therapy.

Alterations in epigenetic modifications such as DNA methylation, histone acetylation and RNA interference are important heritable contributory factors in tumor development [6]. For example, altered DNA methylation is thought to contribute to the pathogenesis of a variety of cancers, including HCC [7]. However, multiple studies indicate that a variety of genes are aberrantly hyper- or hypomethylated in HCC [8], but a comprehensive profile of the pathways within the interaction network remains to be elucidated. Several genes encoding epigenetic regulatory proteins, including EZH2 and HBV, have been shown to be involved in hepatocellular malignancy [9,10]. In addition, evidence now suggests that methylated mRNA may be a valid predictor of HCC [11]. But to the best of our knowledge, there are no prior studies examining methylated differentially expressed genes (MDEGs) on a genome-wide scale and focusing on predicting prognosis in HCC. In the present study, therefore, we comprehensively analyzed high-dimensional data from the Gene Expression Omnibus (GEO) and The Cancer Genome Atlas (TCGA) to build a novel MDEG-based risk score system that is predictive of prognosis and could potentially guide personalized therapy for HCC patients.

## RESULTS

### Identification and enrichment analysis of MDEGs in HCC

**Figure 1:**
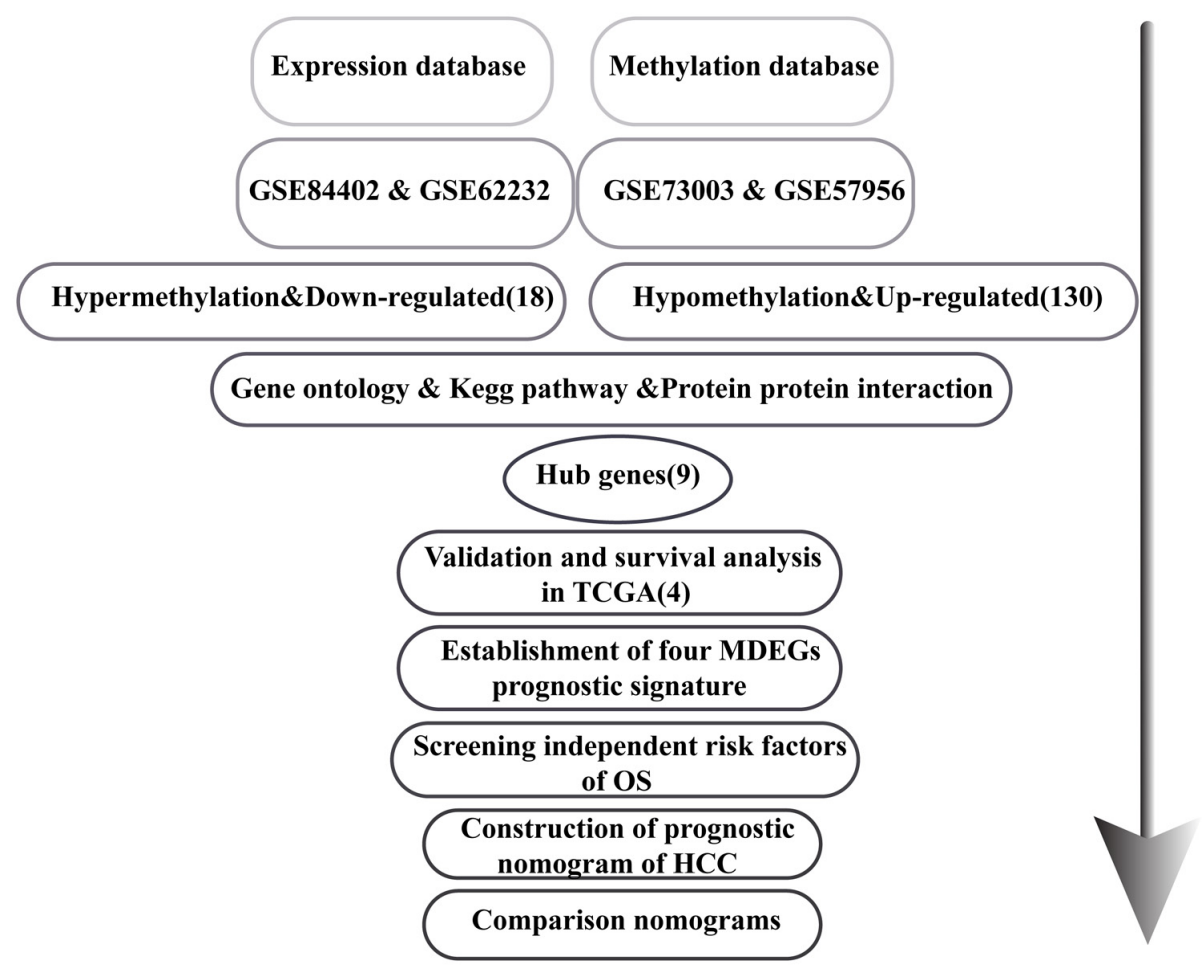
Flowchart of the study.

**Figure 2:**
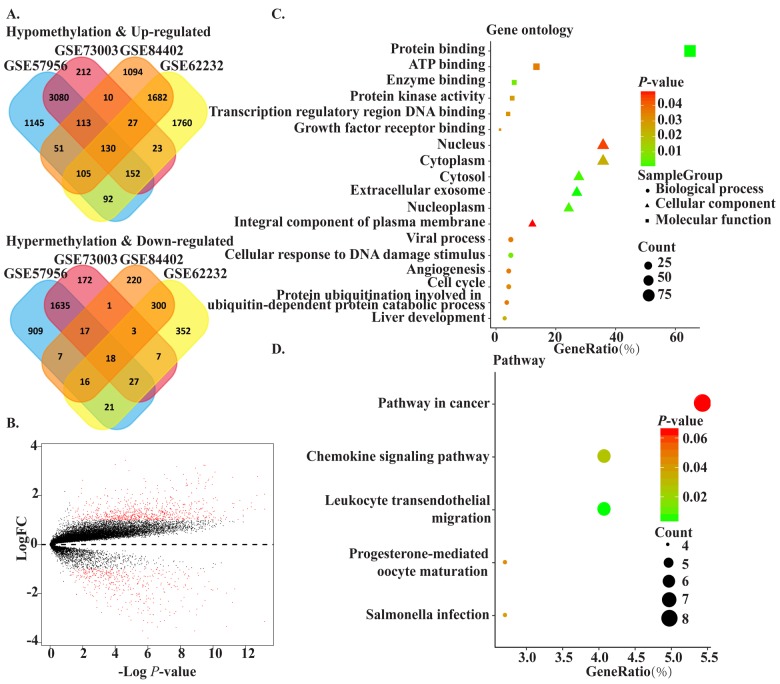
**The methylated-differentially expressed genes identification and function.** (**A**) Venn of methylated-differentially expressed genes in gene expression datasets (GSE62232, GSE84402) and gene methylation datasets (GSE73003, GSE57956). (**B**) The volcano plot of GSE84402. Log2 (FC) vs. -log10 (p value) for differentially expressed mRNA. Red dot represents significant mRNA (log2|FC|>1, P<0.05). (**C**) The significant enriched gene ontology of MDEGs. (**D**) The significant enriched KEGG pathways of MDEGs.

The flowchart for this study is shown in Figure 1. GSE62232, GSE84402, GSE73003 and GSE57956 comprised the training cohort downloaded from the GEO database. The mRNA expression datasets GSE62232 and GSE84402 were calculated using the limma package in R (v 3.5.1). GSE62232 included 81 HCC and 10 normal liver samples, while, GSE84402 included 14 paired HCC and non-tumor samples (Affymetrix Human Genome U133 plus 2.0 platform). The GEO2R online analysis tool was used to calculate the datasets for the methylation difference profiles GSE73003 and GSE57956. The GSE73003 series consisted of 20 paired HCC and non-tumor samples, while GSE57956 consisted of 59 paired HCC and non-tumor samples (Illumina Human Methylation27 BeadChip). With cut-off criteria of *P* < 0.05 and |log2FC| > 1, a total of 130 hypomethylation-high expression genes were detected by overlapping 3476 hypomethylated genes (4869 in GSE57956, 3748 in GSE73003) and 1945 upregulated genes (3972 in GSE62232, 3213 in GSE84402). Similarly, 18 hypermethylation-high expression genes were detected by overlapping 1689 hypermethylated genes (2651 in GSE67956, 1881 in GSE73003) and 338 downregulated genes (583 in GSE62232, 745 in GSE73003) (Figure 2A). To confirm that the *P* value and |log2FC| conform to logic using a different test, a representative volcano plot was constructed for GSE84402 (Figure 2B).

To obtain a deeper understanding of MDEGs, enrichment analysis with the Database for Annotation, Visualization and Integrated Discovery (DAVID, https://david.ncifcrf.gov/) was used to elucidate biological function. The top significant terms emerging form the gene oncology enrichment analysis are shown in Figure 2C. MDEGs were enriched in “biological processes of cellular response to DNA damage stimulus,” “liver development,” “viral process,” “angiogenesis,” and “cell cycle.” Regarding molecular function, MDEGs showed enrichment in “protein binding,” “ATP binding,” “enzyme binding,” and “protein kinase activity.” Enrichment of cell components was mostly “nucleus region,” which suggests MDEGs may play an important role in transcription in HCC. Kyoto Encyclopedia of Genes and Genomes (KEGG) analysis suggested that MDEGs were significantly enrichened in pathways in “cancer,” “leukocyte transendothelial migration,” and “chemokine signaling pathway.” (Figure 2D).

### Identification of hub MDEGs and their clinical value in HCC

**Figure 3:**
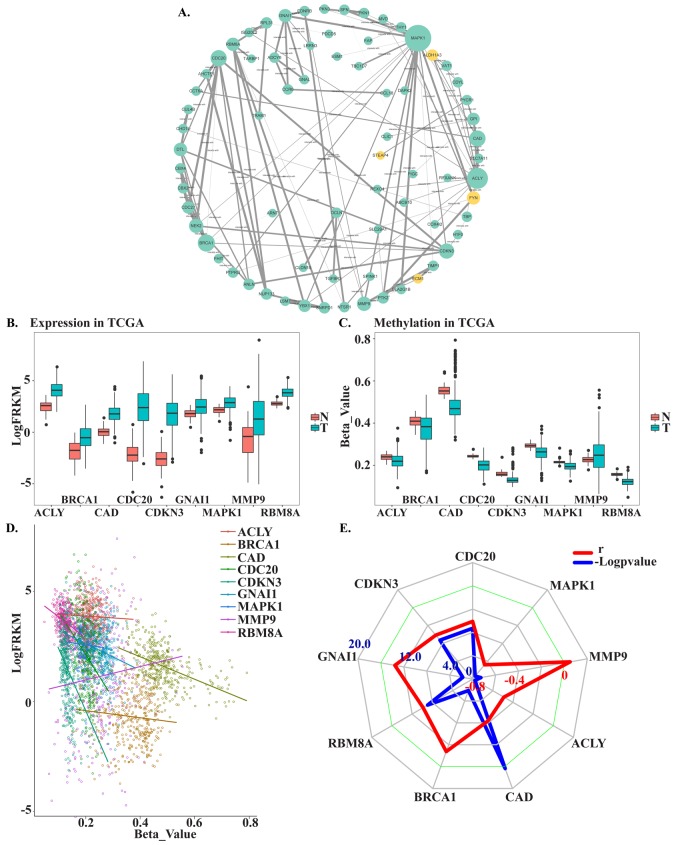
**Screening and verifying hub MDEGs**. (**A**) Protein-protein interaction network of MDEGs. Green dot represents hypo methylation-high expression gene. Yellow dot represents hyper methylation- low expression. The size of dot was decided by the connection degree of gene and the width of line between genes was decided by connectivity between two genes. (**B**) Expression of hub genes in TCGA. (**C**) Methylation of hub genes in TCGA. Beta-Value represents ratio of methylation. T represents tumor tissue, N represents normal tissue. (**D**) Correlation of expression and methylation of hub genes. (**E**) Radar map of hub genes correlation. Red line represents r and blue line represents -Logpvalue.

To identify the connections among MDEGs, a protein-protein interaction (PPI) network for MDEGs was constructed using STRING protein databases (Figure 3A). The top hub genes were CDC20, CDKN3, GNAI1, RBM8A, BRCA1, CAD, ACLY, MMP9, and MAPK1 based on a combined score >0.7 and connection numbers >8. To verify the hub genes, 371 HCC and 50 non-tumor samples were downloaded from TCGA as a validation cohort. Within this group, the expression and methylation values of most hub genes were consistent with the training group, with the exception of MMP9 (Figure 3B, C). We then further investigated the association between gene methylation and expression. The results showed a mild or moderate negative correlation, which suggests methylation leads to decreased gene expression (Figure 3D-E). Expression of CDC20, CDKN3, GNAI1, RBM8A, BRCA1, and CAD showed a significant negative correlation with expression (p<0.05), whereas expression of ACLY, MMP9 and MAPK1 showed no correlation or was positively correlated. And results also were verified in MethHC, a database of DNA Methylation and gene expression in Human Cancer (http://methhc.mbc.nctu.edu.tw/php/index.php) (Supplementary Figure 1).

**Figure 4:**
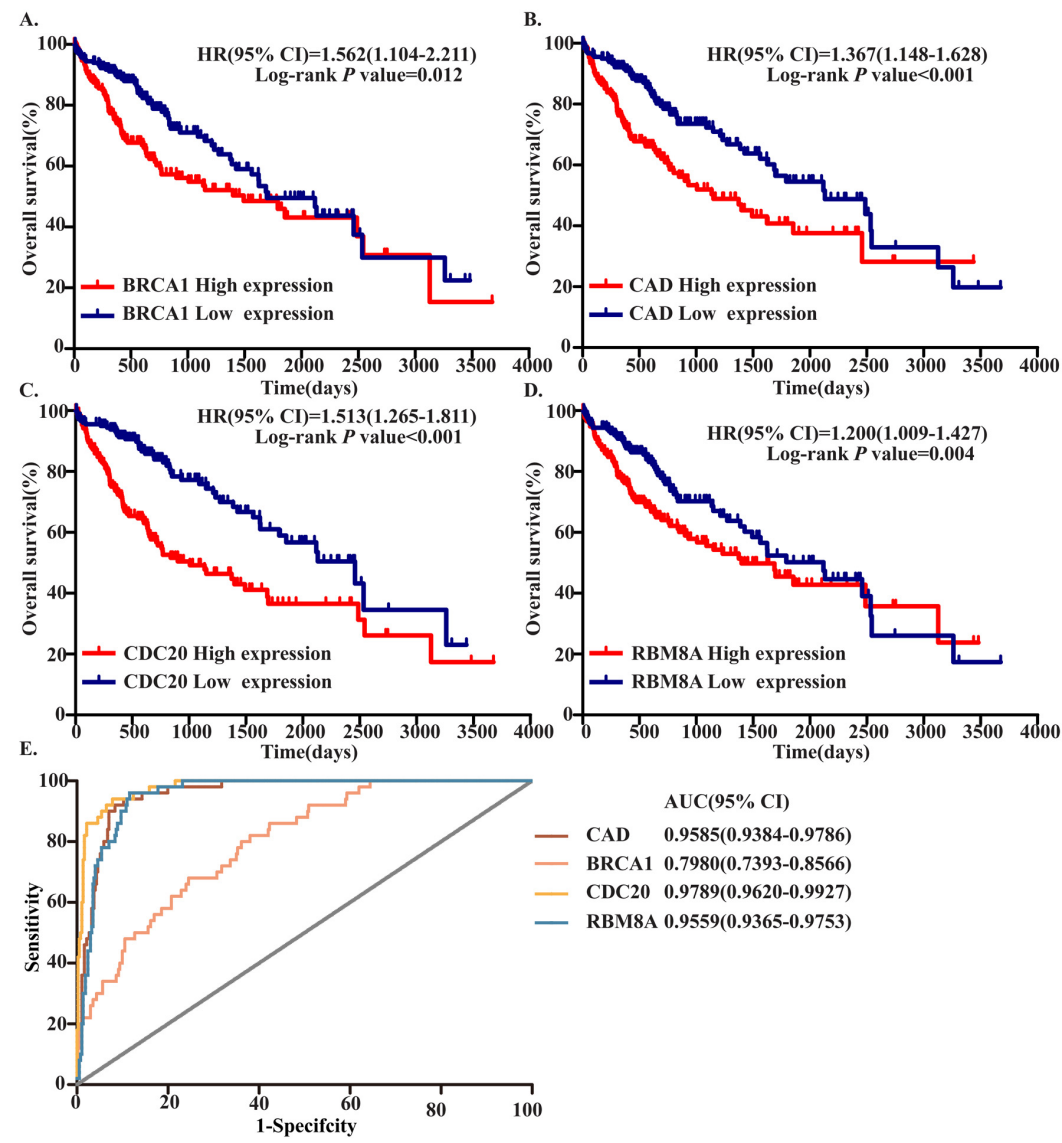
**Four hub MDEGs were associated with overall survival in HCC patients by using Kaplan-Meier curve and Log-rank test.** The patients were stratified into high expression group and low expression group according to median expression of each mRNA. (**A**) BRCA1; (**B**) CAD; (**C**) CDC20; (**D**) RBM8A. (**E**) ROC curves of the 4 hub MDEGs in HCC. The X axis shows false positive rate, presented as "1-Specifcity". The Y axis indicates true positive rate, shown as "Sensitivity".

To identify hub MDEGs with potential prognostic value, we used the Kaplan-Meier method with the Log-rank test to evaluate the relation between expression of the aforementioned genes and the patients’ overall survival (OS). Details of the clinical characteristics are presented in Supplementary Table 1. We found that OS was negatively related to expression of CDC20, RBM8A, BRCA1 and CAD, but had no relation with CDKN3 or GNAI1. Ultimately, the top four hub MDEGs were identified: CDC20, RBM8A, BRCA1 and CAD. To further confirm the results, we verified the four hub genes in Gene Expression Profiling Interactive Analysis (GEPIA, http://gepia.cancer-pku.cn/) (Supplementary Figure 2). Receiver operating characteristic curve (ROC) analysis showed that all four of these genes have high sensitivity and specificity, which suggests high diagnostic value for distinguishing HCC patients from healthy individuals (Figure 4E). These four MDEGs may thus be useful as biomarkers for early diagnosis of HCC.

### Prognostic value of a four-MDEG signature risk score in HCC

**Figure 5:**
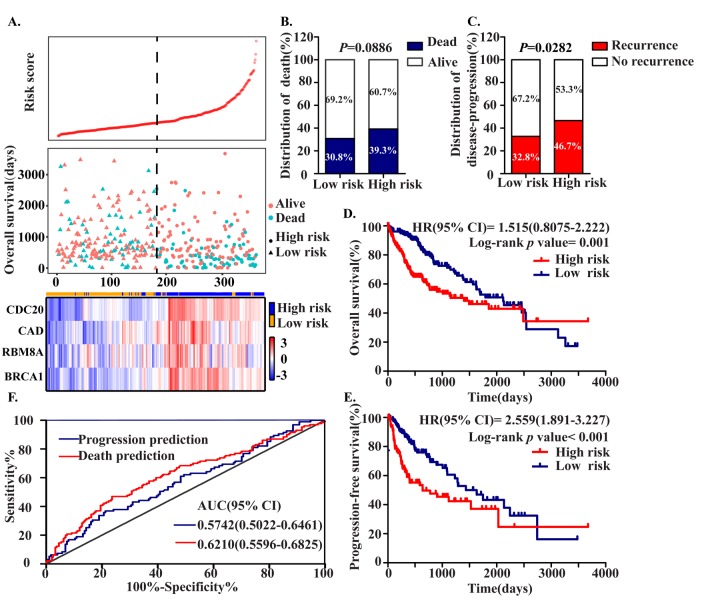
**Construction of the Four MDEGs signature of HCC.** The patients were stratified into high risk group and low risk group based on median of risk score. (**A**) Risk score distribution of HCC patients, Survival status of each patient and Expression heatmap of the four hub MDEGs corresponding to each sample above. Red: high expression; Blue: low expression. (**B**, **C**) The distribution of death (**B**) and disease-progression (**C**) in high and low risk group. (**D**, **E**) Kaplan-Meier estimates of the overall survival (**D**) and progression-free survival (**E**) time of patients using the four MDEGs signature based risk score. (**F**) The ROC curve of the four MDEGs signature.

To assess the prognostic value of CDC20, RBM8A, BRCA1 and CAD, we constructed a prognostic signature by integrating the expression of these four MDEGs using a regression coefficient. We then calculated a risk score for each patient and ranked them based on increasing score, after which patients were classified into a high-risk (n = 179) or a low risk (n = 179) group based on the median risk score. The risk score distribution, survival status, and expression profile of the four prognostic MDEGs are shown in Figure 5A. OS and progression-free survival (PFS) rates among patients were 60.7% and 53.3%, respectively, in the high-risk group, as compared to 69.2% and 67.2% in the low-risk group (Figure 5B, C). The hazard ratio (HR) of high-risk group versus low-risk group was 1.515 for OS (*P* = 0.001, 95% confidence interval (CI) = 0.8075-2.222) and 2.559 for PFS (*P* < 0.001, 95%CI = 1.891-3.227). Thus, patients in the high‑risk group had significantly poorer OS and PFS than patients in the low-risk group (Figure 5D, E).

A ROC analysis of the predictive efficiency of the four-MDEG signature suggested it had good performance with respect to both death and progression prediction (Figure 5F). Taking into consideration the patients’ clinical features, including age, gender, clinical stage, T stage, grade, adjacent hepatic tissue inflammation, and HCC risk factors (virus infection, alcohol abuse, non-alcoholic fatty liver disease, hemochromatosis, alpha-1 antitrypsin deficiency), univariate and multivariate Cox regression analysis were used to assess the signature (high-risk vs. low-risk) with respect to OS. In the univariate analysis, clinical stage (HR = 2.229, *P* < 0.001), T stage (HR = 2.534, *P* < 0.001), HCC risk factors (HR = 0.631, *P* = 0.011), fibrosis (HR = 0.542, *P* = 0.002) and the four-MDEG signature (HR = 4.467, *P* < 0.001) were all significantly associated with OS in HCC patients. To integrate all independent risk factors affecting OS for construction of a HCC prognostic nomogram, significant clinicopathological factors from the univariate analyses were entered into multivariate COX regression analyses. The results indicated that the four-MDEG signature (HR = 2.022, P < 0.001) was a significant independent factor of OS, as were T stage (H = 2.149, *P* < 0.001) and HCC risk factors (HR = 0.651, *P* = 0.019) (Table 1).

**Table 1:** Univariate/multivariate COX regression analyses of clinicopathologic factors associated with OS.

**Variables**	**Univariate analysis**		**Multivariate analysis**	
	**HR (95%CI)**	***P ***	**HR (95%CI)**	***P***
Age (≥65 vs.<65)	1.265(0.893-1.791)	0.235		
Gender (Male vs. Female)	0.817(0.573-1.164)	0.262		
Clinical stage ( III +IV vs. I+II)	2.229(1.559-3.188)	<0.001*		
Grade (G3+G4 vs. G1+G2)	1.113(0.774-1.601)	0.564		
T stage (T3+T4 vs.T1+T2)	2.534(1.783-3.601)	<0.001*	2.149(1.499-3.081)	<0.001*
AFP (<25ng/ml vs. >=25ng/ml)	1.002(0.697-1.442)	0.991		
Adjacent hepatic tissue inflammation ( Yes vs. No)	0.699(0.468-1.044)	0.699		
Fibrosis (Yes vs. No)	0.542(0.366-0.803)	0.002*		
Child-Pugh (A vs. B+C)	1.141(0.578-2.251)	0.703		
BMI (>=25 vs <25)	0.733(0.515-1.043)	0.084		
Family history (Yes vs. No)	1.225(0.858-1.748)	0.264		
HCC risk factors (Yes vs. No)	0.631(0.443-0.898)	0.011*	0.651(0.454-0.933)	0.019*
Four MDEGs signature (high risk vs. low risk)	4.467(1.995-10.002)	<0.001*	2.022(1.486-2.753)	<0.001*

Abbreviations: OS, overall survival; HR, hazard ratio; 95% CI, 95% confidence interval.

*Statistically significant; AFP, Alpha‐fetoprotein; BMI, body mass index.

### Establishment of a nomogram for OS prediction in HCC

**Figure 6:**
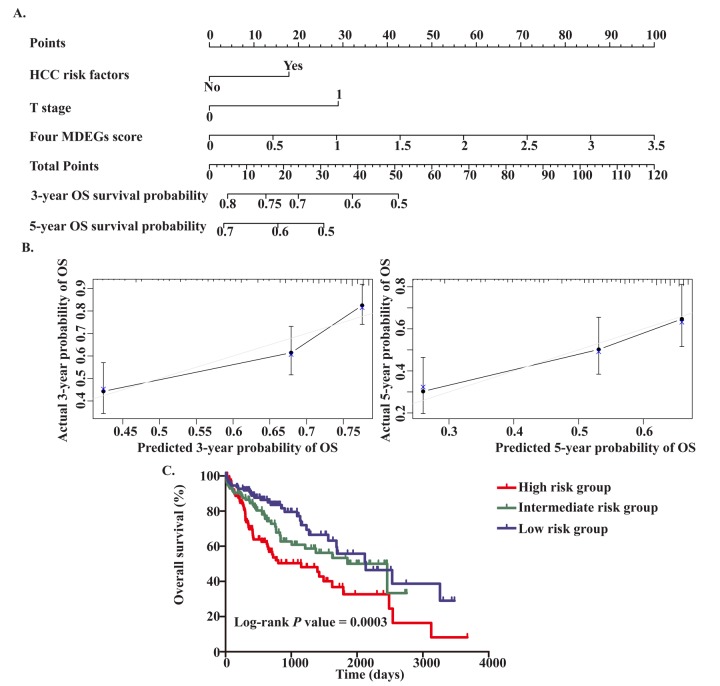
**Establishment of the OS nomogram for HCC patients.** (**A**) Nomogram for predicting OS of HCC. There are three components in this nomogram: the four MDEGs score, HCC risk factor and T stage. Each of them generates points according to the line drawn upward. And the total points of the three components of an individual patient lie on "Total Points" axis which corresponds to the probability of 3‐year and 5‐year OS rate plotted on the two axes below. (**B**) Calibration plots of the nomogram for predicting OS rate at 3 year (Left) and 5 years (Right). The predicted and the actual probabilities of OS were plotted on the x‐ and y‐axis, respectively. (**C**) Kaplan‐Meier curves of three risk subgroups stratified by the total points the nomogram gives.

To provide a clinically associated quantitative method that could be used to predict the probabilities of 3- and 5-year OS in HCC, a prognostic nomogram was established in which the score integrated the three independent prognostic factors, T stage, HCC risk factors and the four-MDEG signature (Figure 6A). Harrel’s concordance index (C-index) for OS prediction was 0.676. The calibration curves for the nomogram for the 3- and 5- year OS rates showed good agreement between the prediction and the actual observation (Figure 6B). Each patient for whom there was complete clinical information about T stage, HCC risk factors, and the four-MDEG signature would obtain a Nomo-score reflecting total points. Using the Nomo-score, patients were divided into three risk groups based on the tertiles, which had cut-off values of 28.50 and 44.60. From KM analysis of the TCGA dataset, significant differences were observed between the high-, intermediate- and low-risk groups (P = 0.0003) (Figure 6C).

### Comparison of predictive accuracy between the nomogram and a single independent factor

The TNM stage system is regarded as the best predictor of survival. Moreover, we found that T stage was an independent prognostic factor for OS in HCC. The predictive power of the nomogram for HCC prognosis was compared with that of T stage. The C-index for OS prediction based on T stage was 0.629, which was significantly lower than the C-index for the nomogram (0.676, P<0.05). This suggests our nomogram is a more accurate predictor of OS in HCC than conventional staging systems and is potentially valuable for predicting survival of HCC patients.

## DISCUSSION

A valid and accurate molecule-based method for identifying patients who have a poor prognosis is urgently needed to optimize their individual therapy. Therefore, effective and credible biomarkers and genetic signatures that can serve as prognostic predictors and treatment targets are critically needed for HCC. In the last decade, methylation has come to be recognized as an important epigenetic regulator of gene expression in eukaryotes, and it is now well established that methylation, especially DNA methylation, is crucially involved in multiple cancers, including HCC [12,13]. Nishida showed that alterations in DNA methylation are a common feature of hepatocarcinogenesis [14]. Although some studies have identified MDEGs in HCC [15,16], their predictive value for HCC patients has not been systematically investigated until now. To our knowledge, this is the first study to develop a MDEG-based risk score that is predictive of prognosis in HCC.

We used methylation and expression microarrays with GEO databases to screen for MDEGs and were able to obtain a set of MDEGs through in *silico* analysis. Enrichment analysis of the MDEGs suggested they were involved in key biological processes, including DNA damage, viral processes, angiogenesis, and cell cycling. Given that hepatitis virus is a main cause of HCC, the presence of DNA damage due to integration of the virus genome into the host DNA is reasonable [17]. Also reasonable is the involvement of angiogenesis, since HCC is a highly vascular tumor. In addition, KEGG enrichments suggested that significantly enriched pathways include chemokine signaling pathways, which suggests that inflammation and immunity are critical factors in the pathogenesis, progression and metastasis of HCC [18]. Chemokine signaling reportedly influences HCC invasion and/or metastasis through effects on the tumor microenvironment [19,20]. Evidence indicates, for example, that chemokines such as CCL5, CCL7, CXCL8 act via CCRs on myeloid-derived suppressor cells (MDSCs) to form an inhibitory tumor microenvironment that promotes tumor pathogenesis, progression and resistance [21–23].

Based on PPI analysis, we identified nine hub genes, which were verified and analyzed in a validation cohort from TCGA. Four of these hub genes, which were significantly associated with OS of HCC patients, were ultimately selected. Earlier research has consistently demonstrated that upregulation of BRCA1, CDC20, RBM8A and CAD promotes progression, invasion, metastasis and chemoresistance in HCC [24–27]. While the efficacy of any single marker is limited, a multiple-marker signature could have greater diagnostic and prognostic value. We therefore constructed a four-MDEG signature that was an independent prognostic factor for HCC patients. This signature was predictive of both OS and PFS. If applied, relatively minor examination using our risk score system could help identify high- and low-risk HCC patients and provide useful information that could aid in selecting a therapeutic strategy.

To increase the accuracy of the prediction of prognosis, both genetic and clinically-related variables were integrated into the nomogram. Ultimately, the OS nomogram included the four-MDEG signature, T stage and HCC risk factors. The nomogram for HCC performed well when used to predict OS, and its predictive ability was verified using a C-index and a calibration curve. Indeed, the nomogram provides greater predictive accuracy for OS than traditional systems. As regards the prognostic signature or nomogram, if we are able to put it into clinical practice in the future, we anticipate being able to identify patients at high-risk of cancer-related death before treatment, and recommend a more aggressive therapeutic strategies with dynamic surveillance. However, there are limitations to our study. First, whether the prognostic signature or nomogram can be applied to patients must be confirmed in larger groups of HCC patients. Second, the molecular mechanism of the four MDEGs in HCC remains to be explored further.

In summary, we have developed a novel four-MDEG expression-based risk score system for objectively and accurately predicting survival and prognosis in HCC patients. In addition, the MDEG signature could also shed new light on the role of methylation in the pathogenesis and progression of HCC, which may provide information helpful for selection of therapeutic strategies. The four MDEGs could potentially serve as biomarkers and therapeutic targets for dynamic surveillance and treatment of HCC patients.

## MATERIALS AND METHODS

### Data processing

The raw data and clinical information were download from the GEO (https://www.ncbi.nlm.nih.gov/geo/) and TCGA (https://cancergenome.nih.gov/). Gene methylation profiling of the GSE73003 and GSE57956 datasets was conducted using the GPL8490 platform (Illumina Human Methylation27 BeadChip), which included 27,578 highly informative CpG sites and more than 14476 genes (http://www.illumina.com/pages.ilmn?ID=243). Gene expression profiling of the GSE84402 and GSE62232 datasets was conducted using the GPL570 platform (Affymetrix Human Genome U133 plus 2.0 Array), which included 54675 unique probes and tested more than 23517 genes (http://www.affymetrix.com/support/technical/byproduct.affx?product=hg-u133-plus). The GSE73003 series consisted of 20 paired HCC and non-tumor samples. The GSE57956 series consisted of 59 paired HCC and non-tumor samples. The GSE84402 series included 14 paired HCC and non-tumor samples. And the GSE62232 series included 81 HCC and 10 normal liver samples. From TCGA, we downloaded 371 HCC and 50 non-tumor samples. The mRNA-seq data were preprocessed and submitted for analysis as the upper quantile normalized FPKM values. GEO2R was used to screen for genes differentially methylated between tumor and non-tumor samples. The differentially expressed genes were identified using the limma package in R. Values of P<0.05 and |FC|≥1 were considered significant. Finally, hypomethylation-high expression genes were detected by overlapping hypomethylated and upregulated genes; similarly, hypermethylation-low expression genes were detected by overlapping hypermethylated and downregulated genes.

### Functional and pathway enrichment analysis

Functional annotations in MDEGs were done using The Database for Annotation, Visualization and Integrated Discovery (DAVID; https://david.ncifcrf.gov/), which enriched gene oncology and pathways. Gene oncology involved three categories: cellular components, molecular function, and biological processes. Pathway enrichment was carried out using the Kyoto Encyclopedia of Genes and Genomes (KEGG, https://www.kegg.jp/), which contains information about genomes, biological pathways, diseases, and chemical substances. The criterion for significant enrichment of biological processes and pathways was *P* = 0.05.

### Hub MDEG screening and verification

STRING protein databases (https://string-db.org/) were used to evaluate interactive relationships among the MDEGs. We used Cytoscape software to construct a network based on the STRING results. Combined scores >0.7 and connection numbers >8 were deemed to indicate hub genes. To confirm the results, the hub MDEGs were validated in TCGA. The Pearson correlation test was used to assess the relationship between hub gene methylation and expression in HCC.

### Formulation of MDEG signatures and association of signatures and clinical features

ROC curve analysis was used to evaluate the diagnostic effectiveness of hub MDEGs. The prognostic value of hub MDEGs were evaluated using the Kaplan-Meier method with the Log-rank test. Hub MDEGs related to OS were considered to be prognostic. Using the combination of weighted MDEG expression values, independent hub MDEG biomarkers were integrated into a MDEG signature using a risk scoring method as shown in the following equation:

$$RiskScore(patient)=\sum_{i=1}^{n}\frac{\text{expression}(mRNA\text{i})}{median}*coefficient(mRNAi)$$

Here, Risk Score (patient) is a MDEG signature risk score for a HCC patient. In addition, *mRNAi* represents the *i*th prognostic mRNA, while expression (*mRNAi*) is the expression value of *mRNAi* for the patient. *Coefficient (mRNAi)* is the regression coefficient of *mRNAi*, which represents the contribution of *mRNAi* to the prognostic risk score. Based on the risk score, patients can be assigned to a high-risk or low-risk group. Subsequently, a risk score system was constructed, and the median risk score was regarded as the cutoff point. HCC patients were then divided into high- and low-risk groups. Kaplan-Meier survival curves were calculated to compare survival and recurrence risk between the high- and low-risk groups.

### Statistical analysis

To identify independent predictors of OS in HCC, univariate Cox regression analysis was performed to evaluate the prognostic value of signatures with a threshold value of 0.2. Multivariate Cox regression analyses were conducted using Forward LR. Hazard ratios (HRs) and 95% confidence intervals (CIs) were computed based on the Cox regression analysis. A nomogram was constructed based on the results of the multivariate Cox regression analyses using rms version 3.5.1 (http://www.r-project.org/). The performance of the nomogram was assessed using Harrel’s concordance index (C-index) and comparing the predicted and actual probabilities for OS. Bootstraps with 1,000 resamples were used for these activities. Comparisons between the nomogram and other staging systems were made using the rcorrp.cens package in Hmisc and were evaluated using the C-index. Each patient received the total points from the nomogram (Nomo-score). KM curve analysis was performed to evaluate the performance of the nomogram by dividing patients into high-, intermediate- and low-risk groups using tertiles of the Nomo-scores as cut-off points. Values of *P* < 0.05 were considered significant. Statistical analysis was performed using the IBM SPSS Statistics software program version 22.0 (IBM Corp., NY, USA).

## Supplementary Material

Supplementary Figures

Supplementary Table
