# Supplementary material for: A four-methylated mRNA signature-based risk score system predicts survival in patients with hepatocellular carcinoma
Source: Aging (Albany NY). 2019 Jan 10;11(1):160–73. doi: 10.18632/aging.101738 (PMC6339794; doi:10.18632/aging.101738)
Supplement: Supplementary Figures [file aging-11-101738-s001.pdf]

SUPPLEMENTARY FIGURES

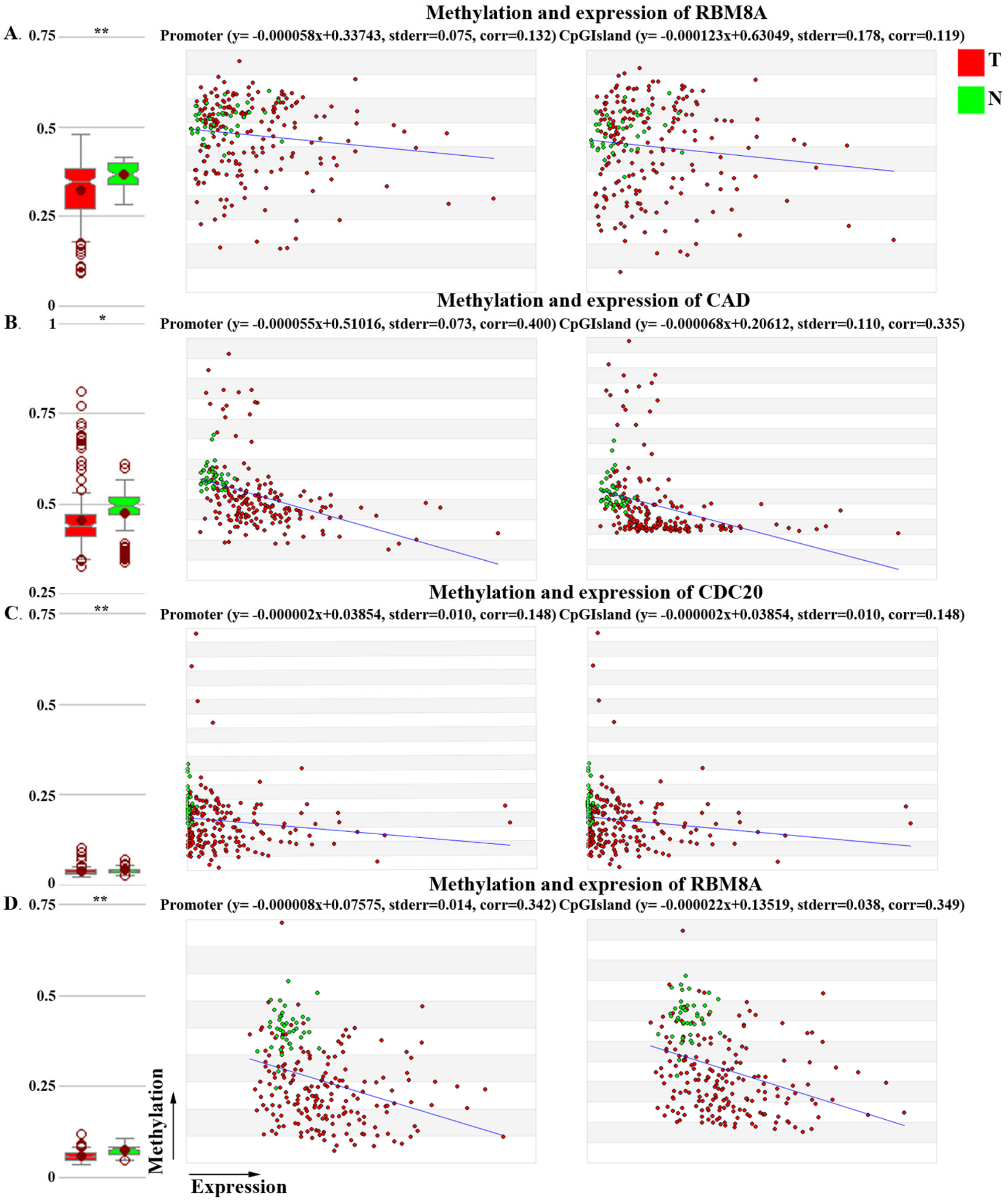

Supplementary Figure 1. Methylation and correlation with expression of the four hub genes. (**A**) BRCA1 (**B**) CAD (**C**) CDC20 (**D**) RBM8A Red dot represents tumor sample; green dot represents normal sample. Horizontal axis is expression and vertical axis is methylation. *: P<0.05; **: P<0.005.

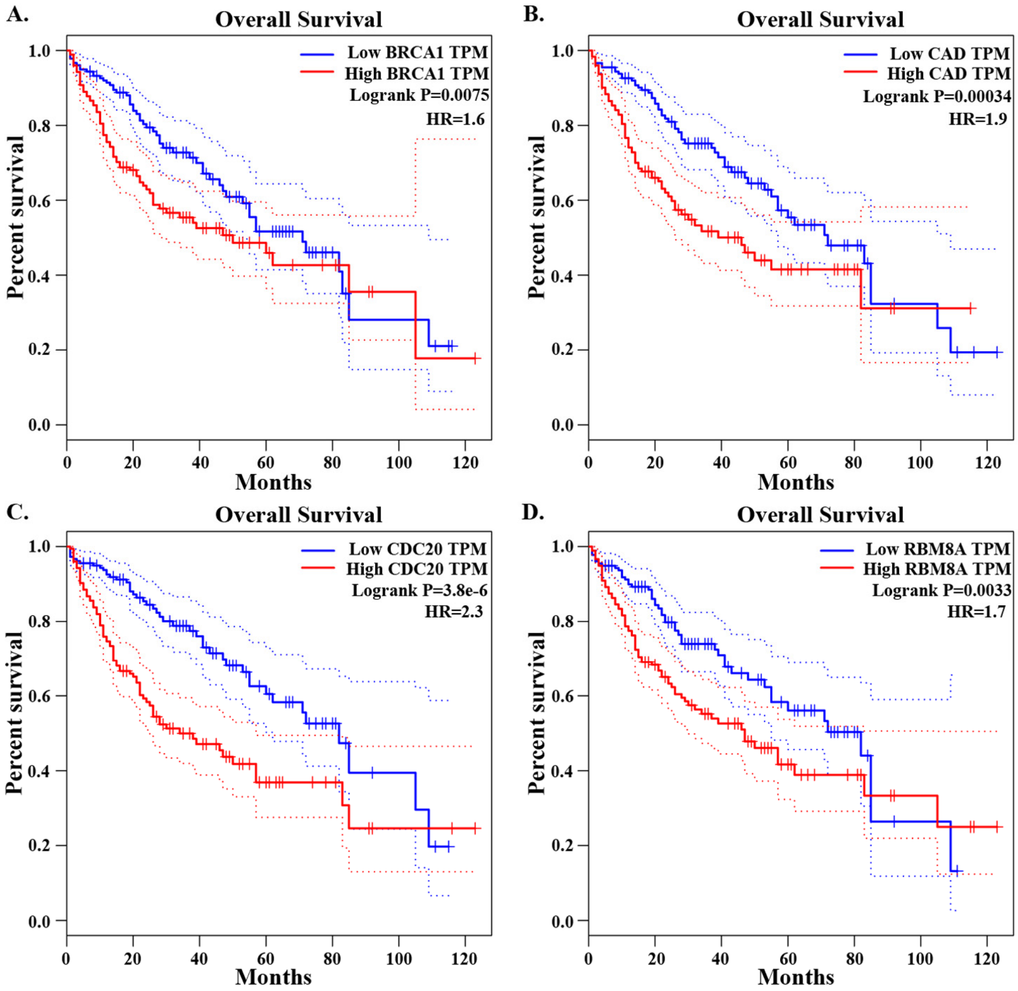

Supplementary Figure 2. Relation between expression of the four hub genes and OS. (**A**) BRCA1 (**B**) CAD (**C**) CDC20 (**D**) RBM8A.
